# Enhanced hemodynamic stability and patient satisfaction with ciprofol-remifentanil versus propofol-remifentanil for sedation in shorter-duration fiberoptic bronchoscopy: a prospective, randomized, double-blind study

**DOI:** 10.3389/fmed.2025.1498010

**Published:** 2025-05-19

**Authors:** Jia Nie, Yongguo Zhang, Yan Wang, Liang Fang, Huanhuan Ma, Yu Zhang, Hai-ying Wang

**Affiliations:** ^1^Department of Anesthesiology, Affiliated Hospital of Zunyi Medical University, Zunyi, China; ^2^Department of Anesthesiology, Guizhou Qiannan People's Hospital, Zunyi, China; ^3^Department of Anesthesiology, Affiliated Xinqiao Hospital of Army Military Medical University, Chongqing, China; ^4^Department of Anesthesiology, Zunyi Maternal and Child Health Hospital, Zunyi, China

**Keywords:** propofol, fiberoptic bronchoscopy, sedation, hemodynamic, ciprofol

## Abstract

**Objective:**

This study aimed to compare the efficacy and safety of ciprofol-remifentanil versus propofol-remifentanil in patients undergoing fiberoptic bronchoscopy (FOB).

**Methods:**

In this prospective, randomized, double-blind, non-inferiority trial, 209 patients undergoing FOB were enrolled and equally divided into two groups (*n* = 106 each). The trial was registered in the Chinese Clinical Trial Registry (ChiCTR) under the registration number ChiCTR2400081603. Patients in the ciprofol-remifentanil group received ciprofol at a dose of 0.4 mg/kg, while those in the propofol-remifentanil group received propofol at a dose of 2.5 mg/kg. Both groups were pre-medicated with 1 μg/kg of remifentanil. Anesthesia was maintained with additional doses of the respective anesthetic agent as required to achieve a Modified Observer’s Assessment of Alertness and Sedation (MOAA/S) scale score of ≤1. The primary outcome was the successful completion rate of FOB. Secondary outcomes included hemodynamic stability, incidence of adverse events such as hypoxemia and hypotension, patient and physician satisfaction, and the incidence of pain on injection.

**Results:**

The successful completion rate of FOB was 92.45% (98 of 106) in the ciprofol-remifentanil group and 90.57% (96 of 106) in the propofol-remifentanil group (*p* > 0.05). The ciprofol-remifentanil group demonstrated more stable hemodynamics, with significantly lower incidences of hypotension and hypoxemia compared to the propofol-remifentanil group (*p* < 0.05). Patient and physician satisfaction scores were significantly higher in the ciprofol-remifentanil group (*p* < 0.05). Additionally, the incidence of pain on injection was significantly lower in the ciprofol-remifentanil group (*p* < 0.01). Other adverse events, including coughing severity and intraoperative awareness, were similar between the two groups (*p* > 0.05).

**Conclusion:**

Ciprofol-remifentanil was non-inferior to propofol-remifentanil in terms of sedation during fiberoptic bronchoscopy;. Furthermore, ciprofol-remifentanil was associated with greater hemodynamic stability, reduced pain on injection, and higher satisfaction scores, suggesting that it may be a preferable alternative to propofol-remifentanil for FOB procedures.

**Clinical trial registration:**

https://www.chictr.org.cn/, ChiCTR2400081603.

## 1 Introduction

The increasing demand for minimally invasive procedures in respiratory medicine has led to the widespread adoption of fiberoptic bronchoscopy (FOB) for the diagnosis and treatment of pulmonary conditions. Traditionally, FOB was performed under local anesthesia, with patients often experiencing significant discomfort, anxiety, and adverse physiological responses such as coughing and hypoxemia. The advent of “painless bronchoscopy,” wherein patients are sedated using intravenous anesthetics, has significantly improved the patient experience and procedural success (1). Among the anesthetics used, propofol has been the cornerstone due to its rapid onset and recovery characteristics. However, propofol’s narrow therapeutic window, potential for significant cardiovascular and respiratory depression, and the absence of a suitable reversal agent have prompted the exploration of alternatives that can provide a more favorable safety profile without compromising efficacy (2).

Ciprofol (HSK3486) is a novel 2,6-disubstituted phenol derivative that acts as a positive allosteric modulator of the GABAA receptor, similar to propofol. Developed to address the limitations associated with propofol, ciprofol has demonstrated a higher therapeutic index, reduced cardiovascular depression, and a decreased incidence of injection pain (3, 4). Recent studies have shown that ciprofol achieves comparable sedation and anesthetic effects at one-fourth to one-fifth the dose of propofol, with a significantly wider safety margin (5). In the context of procedural sedation, ciprofol has been investigated in various clinical settings, including gastrointestinal endoscopy and minor surgeries, where it has shown promise due to its rapid onset, stable hemodynamic profile, and minimal respiratory depression (6).

Several studies have specifically explored the application of ciprofol in FOB. For instance, a recent randomized, double-blind, non-inferiority trial compared the efficacy and safety of ciprofol-remifentanil with the standard propofol-remifentanil regimen during FOB. The results indicated that ciprofol was non-inferior to propofol, with patients experiencing similar levels of sedation and procedural success (7). Additionally, the study highlighted ciprofol’s potential advantages, such as fewer incidences of hypotension and respiratory complications, making it a viable alternative in FOB. However, despite these encouraging findings, the existing research primarily focuses on non-inferiority comparisons and general safety profiles. There has been limited exploration into the detailed hemodynamic effects of ciprofol throughout different stages of the procedure, nor have studies extensively compared patient and physician satisfaction or the incidence of specific adverse events like hypoxemia and post-procedural discomfort. Furthermore, the optimal dosing strategies for ciprofol in combination with remifentanil, particularly in comparison to propofol, remain underexplored.

Our study aims to provide a more comprehensive comparison between ciprofol-remifentanil and propofol-remifentanil during FOB. Specifically, we seek to determine whether ciprofol can offer superior hemodynamic stability, reduce the incidence of hypoxemia and hypotension, and improve overall patient and physician satisfaction. By focusing on these aspects, our research intends to refine the sedation protocols for FOB, potentially leading to safer and more effective anesthesia practices. The study’s findings have the potential to inform clinical practice by identifying an optimized anesthetic protocol that enhances patient safety and comfort while maintaining procedural efficacy.

## 2 Methods

### 2.1 Study design and setting

This prospective, randomized, double-blind, controlled trial was conducted at two centers: Affiliated Hospital of Zunyi Medical University and Qiannan Buyei and Miao Autonomous Prefecture People’s Hospital. The trial was registered in the Chinese Clinical Trial Registry (ChiCTR) under the registration number ChiCTR2400081603. The study was conducted over a 6 months period from March 2024 to August 2024, with Affiliated Hospital of Zunyi Medical University recruiting 152 participants from March 20, 2024, to June 7, 2024, and Qiannan People’s Hospital enrolling 60 participants from July 5, 2024, to August 15, 2024. The study aimed to compare the efficacy and safety of ciprofol-remifentanil (C group) versus propofol-remifentanil (P group) in patients undergoing painless FOB.

### 2.2 Study objectives

The primary objectives of this study were:

1. To determine whether ciprofol combined with remifentanil provides more stable hemodynamics during anesthesia induction and throughout the FOB procedure compared to propofol combined with remifentanil.
2. To assess whether ciprofol combined with remifentanil reduces the incidence and severity of hypoxemia and hypotension during the procedure.

### 2.3 Patients

This trial was approved by the institutional review boards of both hospitals, and written informed consent was obtained from all patients representatives prior to participation. The trial adhered to the principles of the Declaration of Helsinki and Good Clinical Practice guidelines.

At Zunyi Medical University Affiliated Hospital, 152 patients were recruited from March 20, 2024, to June 7, 2024. At Qiannan People’s Hospital, 60 patients were enrolled from July 5, 2024, to August 15, 2024. Participants were selected based on the following inclusion criteria: patients aged 18 to 64 years, scheduled for painless FOB with sedation, American Society of Anesthesiologists (ASA) physical status I–II, Mallampati score I–II, and oxygen saturation (SpO_2_) > 93% on room air.

Exclusion criteria included a history of severe hepatic or renal dysfunction, coagulation disorders, severe respiratory insufficiency, QTc interval ≥450 ms, recent use of drugs affecting QT interval or cytochrome P450 enzymes, history of alcohol or drug abuse, previous anesthesia incidents, or nasopharyngeal surgery, known allergies to propofol, ciprofol, remifentanil, or other related drugs, body mass index (BMI) > 35 kg/m^2^, difficult airway, pregnancy or lactation, presence of central nervous system diseases, severe hypertension, diabetes, or liver and kidney dysfunction, refusal to sign consent forms, participation in other clinical trials within 3 months prior to FOB, procedure time >30 min, and inability to communicate or cooperate during the procedure.

### 2.4 Randomization and blinding

Participants were randomly assigned to the C group or the P group using a computer-generated randomization schedule, stratified by center. Allocation was concealed using opaque, sealed envelopes. Both patients and clinical staff, including those administering anesthesia and performing the bronchoscopy, were blinded to group assignments to minimize bias.

### 2.5 Anesthesia and procedure protocol

All participants fasted for at least 8 h and refrained from drinking for 2 h prior to the procedure. Upon arrival at the operating room, standard monitoring was established, including electrocardiography (ECG), non-invasive blood pressure (NIBP), heart rate (HR), pulse oximetry (SpO_2_), and respiratory rate (RR). Intravenous access was secured, and patients received 2% lidocaine nebulization 30 min before the procedure.Oxygen supply to the patient was initiated before induction (via face mask at 4 L/min). Continuous oxygen supply was maintained from the start of induction until the end of the procedure (at 6 L/min). If SpO₂ < 90%: the procedure was paused, increase FiO₂ to 100%, and provide assisted ventilation if necessary. During the recovery phase, oxygen supply was continued until the patient was fully awake, gradually reducing the oxygen flow rate.

In the C group, patients received an intravenous bolus of ciprofol at a dose of 0.4 mg/kg combined with remifentanil at 1 μg/kg. In the P group, patients received an intravenous bolus of propofol at a dose of 2.5 mg/kg combined with remifentanil at 1 μg/kg. Both groups used nasopharyngeal airway to maintain spontaneous breathing. Sedation depth was monitored using the Modified Observer’s Assessment of Alertness/Sedation (MOAA/S) scale, aiming to maintain a score of ≤1 throughout the procedure. The Modified Observer’s Assessment of Alertness/Sedation (MOAA/S) scale was used to assess the level of sedation every 30 s (by stimulating the patient’s trapezius muscle) until the patient became unresponsive, indicating successful induction. Subsequently, assessments were conducted every 3 min until the end of the procedure. During the recovery phase, the patient’s name was called in a normal voice. A rapid response from the patient, corresponding to a score of 4–5 on the MOAA/S scale, indicated readiness for discharge from the recovery room. The primary outcome was the hemodynamic stability during FOB. Secondary outcomes included was the successful completion rate of FOB. If necessary, additional boluses of ciprofol (0.1–0.2 mg/kg) or propofol (0.2–0.5 mg/kg) were administered to manage patient movement or coughing.

### 2.6 Procedure and monitoring

FBO was performed by experienced bronchoscopists using a standardized technique. During the procedure, hemodynamic parameters (HR, MAP, SpO_2_, RR) were recorded at predefined time points: pre-administration (T0), after drug administration (T1), after nasopharyngeal tube insertion (T2), following the bronchoscope’s entry into the carina (T3), at the end of the examination (T4), and at awakening (T5).

### 2.7 Outcome measures

Primary outcomes included the hemodynamic stability of patients, assessed by monitoring HR, MAP, SpO_2_, and RR during the procedure. Secondary outcomes included the incidence of adverse events, such as hypoxemia (SpO_2_ < 90%), hypotension (MAP decrease ≥ 20% from baseline), post-procedural discomfort, and injection pain.

Patient and physician satisfaction were assessed using a 100-point scale. Satisfaction levels were categorized as satisfied (≥90), somewhat satisfied (60–89), or dissatisfied (<60). Additional data collected included the time to onset of sedation, time to recovery (MOAA/S = 5), and total drug dosage administered during the procedure.

### 2.8 Sample size calculation

The sample size was calculated based on the primary outcome of oxygen saturation below 93%. According to our pilot study the incidence of hypoxemia was 14% in the intervention group and 34% in the control group. Using a significance level of 0.05 and a power of 80%, it was determined that 72 patients per group were required. Allowing for a 5% dropout rate, the final sample size was set at 76 patients per group, totaling 152 participants across both centers.

### 2.9 Statistical analysis

Statistical analysis was performed using Statistical Package for the Social Sciences version 26.0 (IBM, Armonk, NY). Continuous variables were expressed as mean ± standard deviation (SD) and compared using independent t-tests or Mann–Whitney U tests, as appropriate. Categorical variables were analyzed using chi-square tests or Fisher’s exact test. A *p*-value of <0.05 was considered statistically significant.

### 2.10 Ethical considerations

This study was conducted in accordance with the Declaration of Helsinki and was approved by the ethics committee of Zunyi Medical University Affiliated Hospital and Qiannan People’s Hospital. Written informed consent was obtained from all participants. The trial was registered in the Chinese Clinical Trial Registry (ChiCTR) under the registration number ChiCTR2400081603.

## 3 Results

### 3.1 Patient demographic characteristics

**Figure 1:**
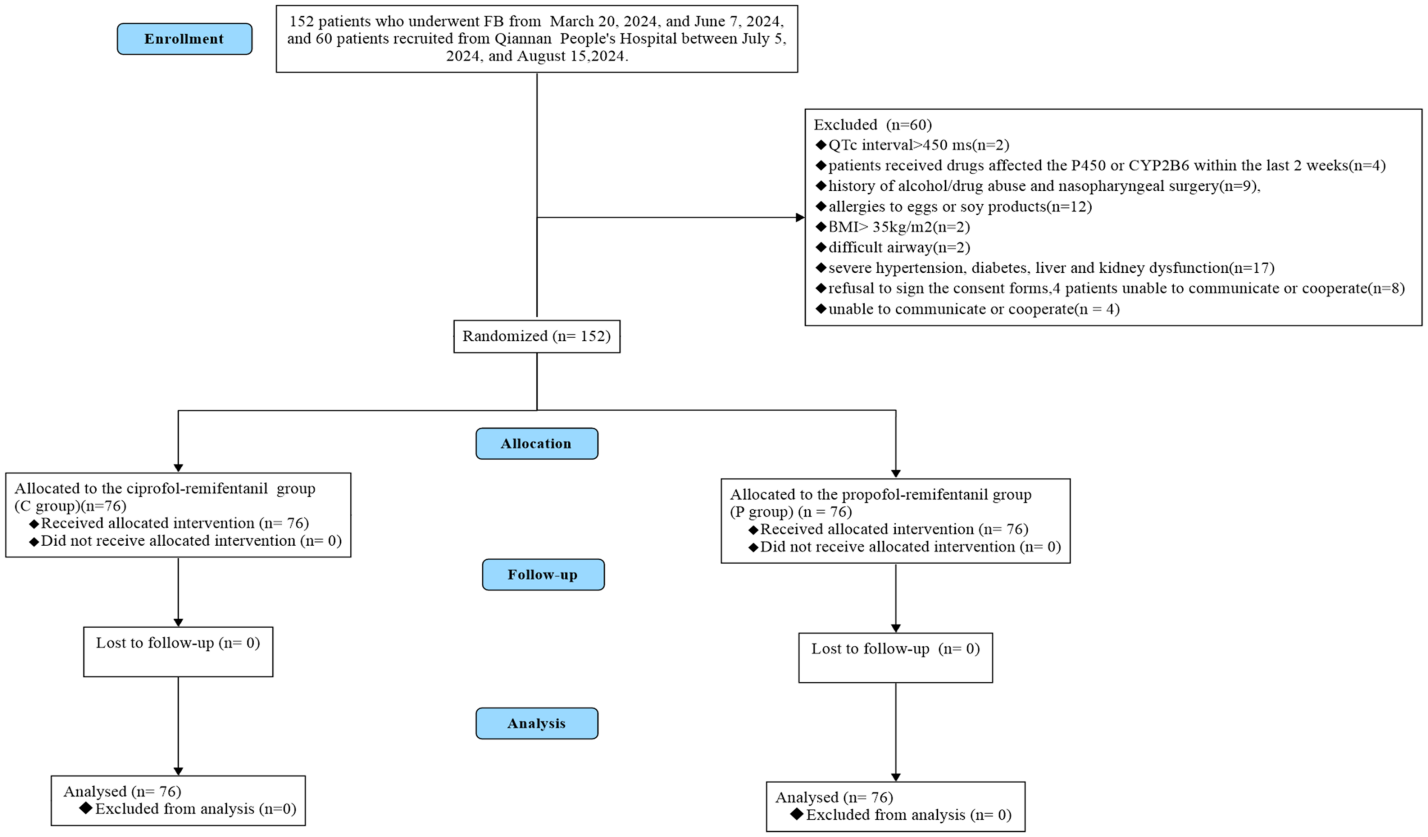
Patient flowchart with CONSORT guidelines.

As illustrated in Figure 1, a total of 209 patients were enrolled in this study, with 152 patients recruited from Zunyi Medical University Affiliated Hospital between March 20, 2024, and June 7, 2024, and 60 patients recruited from Qiannan Buyei and Miao Autonomous Prefecture People’s Hospital between July 5, 2024, and August 15, 2024. Of these, 60 patients were excluded for the following reasons: patients with QTc interval ≥ 450 ms (*n* = 2), received drugs affected the P450 or CYP2B6 within thelast 2 weeks (*n* = 4), history of alcohol/drug abuse and nasopharyngeal surgery (*n* = 9), allergies to eggs or soy products (*n* = 12), BMI > 30kg/m^2^ (*n* = 2), difficult airway (*n* = 2), severe hypertension,diabetes,liver and kidneydysfunction (*n* = 17), patients refusal to sign the consent forms (*n* = 8), patients unable to communicate or cooperate (*n* = 4). Ultimately, 152 patients were randomly assigned to the ciprofol-remifentanil (C) group (*n* = 76) or the propofol-remifentanil (P) group (*n* =  76) The demographic characteristics of patients in both groups were similar, with no significant differences observed (*p* > 0.05; Table 1).

**Table 1:** Basic characteristics of patients.

Characteristics	C group (*n* = 76)	P group (*n* = 76)	*p* value
Gender(%), male/female	46/30	45/31	1.000
Age (IQR), years	56.00 [45.00, 64.75]	56.50 [46.00, 61.00]	0.4180
BMI (mean ± SD)	22.89 ± 2.86	22.04 ± 2.93	0.075
ASA grade (%), I/II	3/72	2/74	0.6810

C group: Cyclopropofol group + Remifentanil; P group: Propofol group + Remifentanil.

### 3.2 Efficacy

#### 3.2.1 Primary outcome

**Figure 2:**
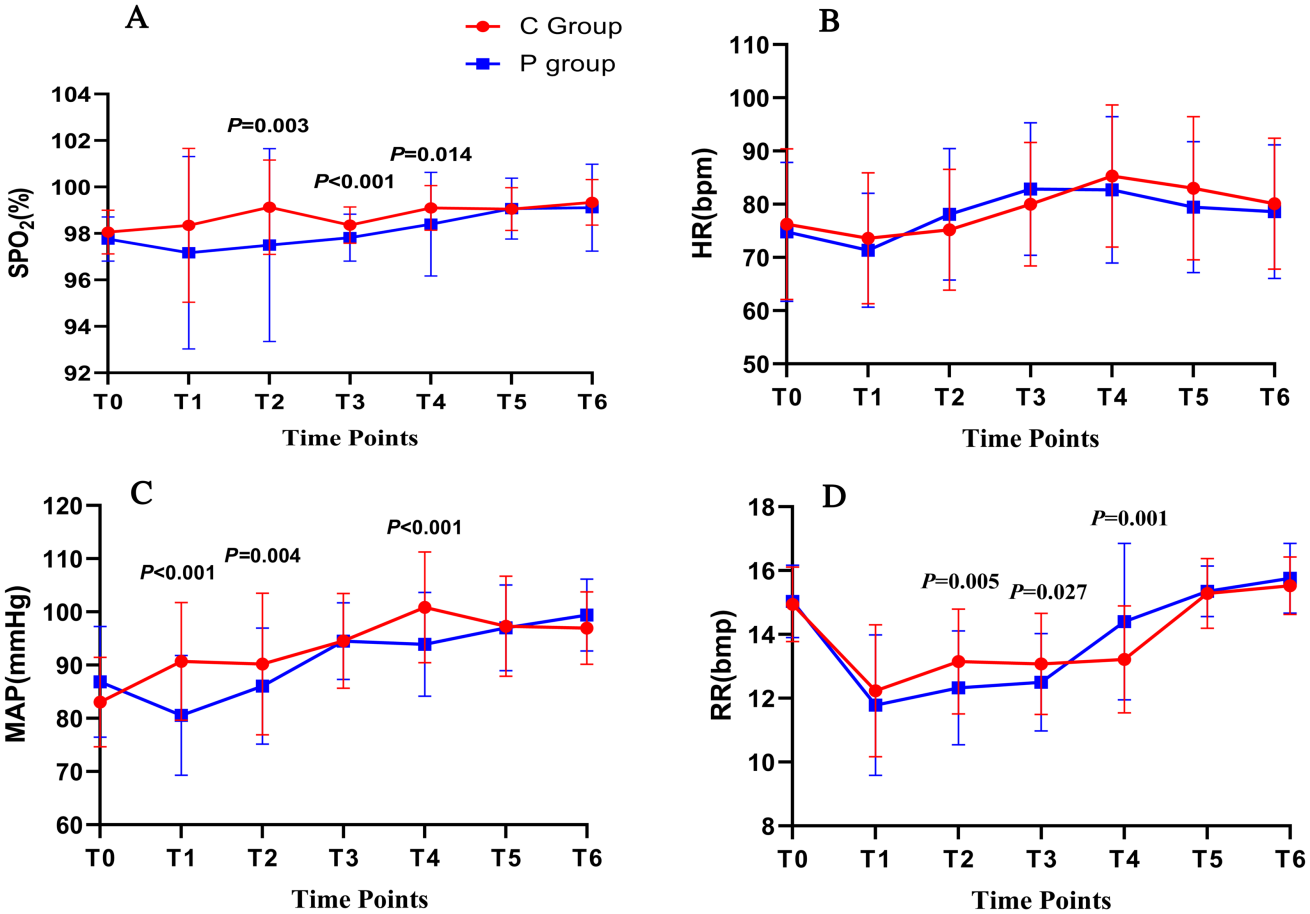
Hemodynamic and respiratory changes during FOB under Ciprofol-remifentanil (C group) and Propofol-remifentanil (P group). **(A)** Oxygen saturation (SpO_2_) over time, showing significantly higher SpO_2_ levels in the C group compared to the P group at T2 (after nasopharyngeal tube insertion), T3 (after bronchoscope enters the carina), and T5 (at the end of the examination). **(B)** Heart rate (HR) remained relatively stable across all time points, with no significant differences between the C and P groups. **(C)** Mean arterial pressure (MAP) showed better stability in the C group, with significantly higher values at T1 (after drug administration), T3, and T4 compared to the P group. **(D)** Respiratory rate (RR) was significantly higher in the C group at T1, T3, and T4, indicating more stable respiratory function in the C group. *p* values indicate significant differences between the C and P groups at specific time points. Error bars represent standard deviations.

The primary outcome was the hemodynamic stability during FOB. Figure 2 presents changes in SpO_2_ (A), heart rate (HR) (B), MAP (C), and respiratory rate (RR) (D) at six time points during the procedure. In panel (A), SpO_2_ values were significantly higher in the C group compared to the P group at T2, T3, and T4 (*p* = 0.029, *p* = 0.002, and *p* = 0.001, respectively). Panel (B) shows that HR remained relatively stable in both groups, with no significant differences observed. However, in panel (C), MAP was more stable in the C group, with significantly better maintenance at T2, T3, and T4 (*p* < 0.001, *p* = 0.004, and *p* < 0.001, respectively). In panel (D), RR was significantly higher in the P group at T2 and T3 (*p* = 0.005 and *p* = 0.027, respectively), though both groups maintained RR within normal ranges throughout the procedure.

The successful completion rates of FOB were 94.7% (89 of 94; 95% CI: 88.5–99.0%) in the C group and 92.7% (89 of 96; 95% CI: 87.0–98.5%) in the P group. The difference in successful completion rates between the two groups was 2.0% (95% CI: 0.5–3.5%), indicating non-inferiority of ciprofol-remifentanil compared to propofol-remifentanil (Table 2).

**Table 2:** Changes in vital signs of patients during medications administration.

Vital signs	C group	P group	*p* value
HR (bpm)			
T0	75.00 [66.00, 88.00]	73.00 [65.25, 82.00]	0.4530
T1	72.00 [65.25, 83.00]	69.00 [63.00, 81.00]	0.1970
T2	75.21 ± 11.35	78.12 ± 12.35	0.1330
T3	80.00 ± 11.61	82.88 ± 12.43	0.1420
T4	85.31 ± 13.45	82.71 ± 13.75	0.2380
T5	83.03 ± 13.45	79.46 ± 12.30	0.0900
T6	80.13 ± 12.28	78.60 ± 12.56	0.4500
MAP (mmHg)			
T0	84.50 [78.25, 88.75]	85.00 [80.00, 92.00]	0.0920
T1	90.72 ± 11.07	80.57 ± 11.26	<0.0001
T2	91.00 [87.00, 96.00]	87.00 [78.25, 94.00]	0.004
T3	94.59 ± 8.93	94.54 ± 7.22	0.9680
T4	105.00 [96.00, 108.00]	96.00 [87.00, 100.00]	<0.0001
T5	99.00 [94.00, 103.00]	99.50 [92.00, 101.00]	0.3920
T6	98.00 [92.00, 101.75]	100.00 [96.00, 104.00]	0.0520
SPO_2_ (%)			
T0	98.00 [97.25, 99.00]	98.00 [97.00, 98.00]	0.0640
T1	100.00 [99.00, 100.00]	99.00 [96.00, 100.00]	0.0290
T2	100.00 [99.00, 100.00]	99.00 [97.00, 100.00]	0.0020
T3	100.00 [99.00, 100.00]	100.00 [99.00, 100.00]	0.9140
T4	100.00 [99.00, 100.00]	99.00 [98.00, 100.00]	0.2320
T5	99.00 [99.00, 100.00]	99.00 [96.00, 100.00]	0.2200
T6	98.00 [98.00, 99.00]	98.00 [98.00, 98.00]	0.0010
RR (bmp)			
T0	15.00 [15.00, 16.00]	15.00 [15.00, 16.00]	0.6050
T1	12.00 [11.00, 14.00]	12.00 [11.00, 13.00]	0.4400
T2	13.00 [12.00, 15.00]	12.00 [11.00,14.00]	0.0050
T3	13.00 [12.00, 14.75]	12.00 [11.25, 13.00]	0.027
T4	13.00 [12.00, 14.00]	15.00 [11.25, 16.00]	0.001
T5	16.00 [15.00, 16.00]	15.00 [15.00, 16.00]	0.4530
T6	15.00 [15.00, 16.00]	16.00 [15.00,1 6.00]	0.0730

T0: Pre-administration; T1: After administration; T2: After asopharyngeal tube insertion; T3: After asopharyngeal tube insertion; T4: After the bronchoscope enters the carina; T5: At the end of the examination; T6: at awakening.

#### 3.2.2 Secondary outcomes

**Figure 3:**
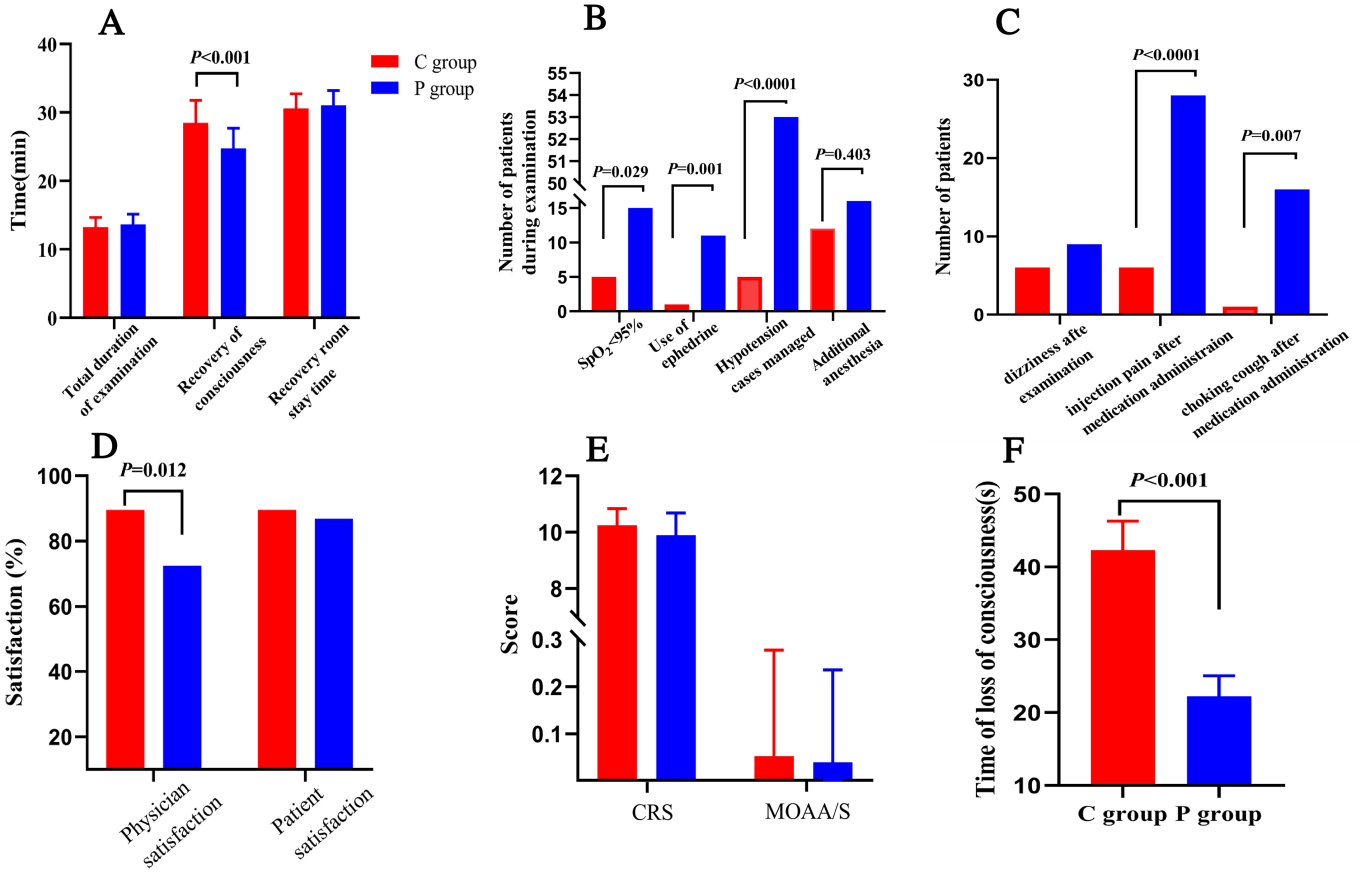
Secondary outcomes and safety parameters in Ciprofol-remifentanil (C group) and Propofol-remifentanil (P group) groups during FOB. **(A)** Time metrics including total examination duration, recovery of consciousness time, and recovery room stay time. Recovery of consciousness was significantly faster in the P group compared to the C group (*p* < 0.001). **(B)** Number of patients with SpO_2_ < 95%, use of ephedrine, and hypotension management. The P group had significantly more patients requiring intervention for hypotension (*p* < 0.0001). **(C)** Incidence of adverse events, including dizziness, injection pain, and choking cough after medication administration. The P group experienced more adverse events compared to the C group, with significant differences in injection pain (*p* < 0.0001) and choking cough (*p* = 0.007). **(D)** Satisfaction scores for physicians and patients. Physician satisfaction was significantly higher in the C group compared to the P group (*p* = 0.012). **(E)** Sedation depth measured by the Clinical Respiratory Score (CRS) and the Modified Observer’s Assessment of Alertness/Sedation (MOAA/S). No significant differences were observed between the groups. **(F)** Time of loss of consciousness was significantly longer in the C group compared to the P group (*p* < 0.001). Error bars represent standard deviations.

The induction time, defined as the time from drug administration to a MOAA/S score of ≤1, was longer in the C group compared to the P group (mean induction time: 3.5 ± 1.5 min vs. 2.8 ± 1.2 min, *p* < 0.05). Recovery time, defined as the time from the end of the procedure to MOAA/S = 5, was significantly shorter in the C group (mean recovery time: 5.4 ± 2.3 min vs. 6.8 ± 2.7 min, *p* < 0.05; Figure 3A). The total procedure duration was comparable between the two groups.

Figure 3B highlights that the P group had a significantly higher number of patients experiencing SpO_2_ < 95% (*p* = 0.029), greater use of ephedrine to manage hypotension (*p* = 0.001), and a significantly higher incidence of hypotension cases managed with intervention (*p* < 0.0001).There was no statistically significant difference in the frequency of additional anesthesia medication administrations between the two groups during the surgery.

Patient and physician satisfaction were assessed using a 100-point scale. Satisfaction scores were higher in the C group compared to the P group (Figure 3D), with more patients in the C group willing to undergo the procedure again under the same anesthesia scheme (91.5% vs. 76.8%, *p* < 0.05). Physician satisfaction was also significantly higher in the C group compared to the P group (*p* = 0.012).

Figure 3E illustrates that the CPS scores for patient consciousness levels during the procedure were comparable between the two groups, while MOAA/S scores were similarly low, reflecting deep sedation achieved in both groups.

Lastly, as shown in Figure 3F, the time to loss of consciousness was significantly shorter in the P group compared to the C group (*p* < 0.001), suggesting a more rapid onset of sedation with propofol. However, despite this, the overall efficacy and safety outcomes favor the C group, particularly regarding recovery time and hemodynamic stability (Figure 3).

### 3.3 Safety

#### 3.3.1 Hemodynamic parameters

Hemodynamic parameters, including HR, MAP, and SpO_2_, were monitored throughout the procedure. Figure 3 shows that HR (Figure 3B) and MAP (Figure 3C) were more stable in the C group, particularly at T3 and T4. SpO_2_ levels (Figure 3A) were significantly higher in the C group compared to the P group at T2, T3, and T4 (*p* < 0.05). Respiratory rate (Figure 3D) was higher in the P group at T2 and T3 (*p* < 0.05).

### 3.4 Adverse events

Figure 3B summarizes the adverse events. Patients in the P group were significantly more likely to experience hypotension (*p* < 0.0001), SpO_2_ < 95% (*p* = 0.029), and require ephedrine administration (*p* = 0.001). The P group also had a higher incidence of dizziness, injection pain, and choking cough after medication administration (Figure 3C), with significant differences observed for injection pain (*p* < 0.0001) and choking cough (*p* = 0.007).

## 4 Summary of findings

Overall, the C group demonstrated a more favorable safety profile, with fewer occurrences of adverse events, better maintenance of hemodynamic stability, and higher patient and physician satisfaction compared to the P group. The induction time was slightly longer in the C group, but recovery times were shorter, and both groups achieved comparable procedure durations (Table 3).

**Table 3:** Comparison of relevant indicators between group C and group P.

Characteristics	C group (*n* = 76)	P group (*n* = 76)	*p* value
Total duration of examination (IQR), min	14.00 [12.00, 14.00]	14.00 [13.00, 14.00]	0.134
Recovery of consciousness (IQR), min	29.00 [26.00, 31.00]	24.00 [23.00, 26.00]	<0.0001
Recovery room stay time (IQR), min	30.00 [30.00, 31.00]	30.00 [30.00, 32.00]	0.119
CRS score (IQR)	10.00 [10.00, 11.00]	10.00 [9.00, 10.00]	0.005
MOAA/S score (mean + SD)	0.0526 ± 0.22478	0.0395 ± 0.196	0.447
Time of loss of consciousness (IQR), sec	42.00 [39.25, 45.00]	22.00[20.00, 24.00]	<0.0001
Number of patients with SpO_2_ < 95% (%), Yes/No	5/71	15/61	0.029
Use of ephedrine (%), Yes/No	0/76	11/65	0.001
Number of hypotension cases Managed during examination (%), Yes/No	5/71	53/23	<0.0001
Times of additional anesthesia administrations (Propofol/Ciprofol) (Yes/No)	12/64	16/60	0.403
Patients with dizziness after examination (%), Yes/No	6/70	9/67	0.588
Patients with injection pain (%), Yes/No	6/70	28/48	<0.0001
T3 patients with choking (%), Yes/No	1/75	16/60	0.007
Physician satisfaction (%), Yes/No	68/8	55/21	0.012
Patient satisfaction (%), Yes/No	68/8	66/10	0.803

Ephedrine is 6 mg each time; CRS, Coma Recovery Scale; MOAA/S, Modified Observer’s Assessment of Alertness/Sedation; T3, After asopharyngeal tube insertion.

## 5 Discussion

This study aimed to evaluate the efficacy and safety of ciprofol-remifentanil versus propofol-remifentanil in providing sedation during FOB. Our findings demonstrate that ciprofol-remifentanil is non-inferior to propofol-remifentanil for successful sedation during FOB. The ciprofol-remifentanil combination was associated with more stable hemodynamic parameters, particularly a lower incidence of hypotension and hypoxemia compared to the propofol-remifentanil group. Additionally, the incidence of pain on injection was significantly reduced in the ciprofol-remifentanil group, and both patient and physician satisfaction scores were higher in this group, suggesting potential benefits of ciprofol as an alternative anesthetic agent for FOB (5, 7).

Since the introduction of propofol in the late 1980s, it has become the standard intravenous anesthetic due to its rapid onset, short recovery time, and favorable pharmacokinetic profile. Propofol’s widespread use is attributed to these characteristics, which make it particularly suitable for short procedures such as FOB (1, 8). However, propofol is not without its drawbacks. It is associated with a relatively narrow therapeutic window, and common adverse effects include pain on injection, significant circulatory and respiratory depression, and, in rare cases, propofol infusion syndrome, particularly with prolonged use (1, 8, 9). These limitations have prompted the search for alternative agents that could offer similar efficacy with improved safety profiles. Moreover, the narrow therapeutic index of propofol necessitates careful titration to avoid adverse effects, which can complicate its use in procedures requiring fine adjustments in sedation depth (10).

Ciprofol, a novel intravenous anesthetic, shares a similar molecular structure with propofol but incorporates an R-configured diastereoisomer and a cyclopropyl group. This structural modification enhances steric effects and introduces stereoselectivity, which may contribute to its improved safety profile (3, 11). Several studies have shown that ciprofol is well-tolerated in various clinical settings, inducing dose-dependent sedation and anesthesia with a rapid onset and recovery profile. Importantly, ciprofol achieves these effects at doses that are only 20–25% of those required for propofol, highlighting its potency (4–6, 11). The lower dosage requirement of ciprofol compared to propofol could be particularly advantageous in reducing the total drug exposure, thereby minimizing the potential for adverse effects such as respiratory depression and hypotension (11).

Our study’s results are consistent with these previous findings, demonstrating that ciprofol is associated with more stable hemodynamics during procedures. Specifically, patients in the ciprofol-remifentanil group experienced fewer fluctuations in MAPand SpO_2_ compared to those in the propofol-remifentanil group. These differences were particularly notable during critical points of the procedure, such as bronchoscope insertion and the end of the examination (12, 13). These findings are consistent with other studies that have reported ciprofol’s superior cardiovascular stability compared to propofol, likely due to its less pronounced effects on peripheral vasodilation and myocardial depression (14). Meanwhile, in the present study, no statistically significant differences in heart rate were observed between the two groups at any time points, which could be attributed to limited intraoperative heart rate fluctuations under standardized anesthesia management. This stability is a critical factor in the context of FOB, where maintaining hemodynamic stability is essential for patient safety, particularly in patients with pre-existing cardiovascular conditions. The reduced impact on cardiovascular parameters could make ciprofol a preferred agent in patients with compromised cardiac function, where the risk of hypotension and bradycardia must be minimized (15).

In addition to its hemodynamic benefits, our study found that the recovery time was shorter in the ciprofol-remifentanil group, despite a slightly longer induction time compared to the propofol-remifentanil group. This could be attributed to the pharmacokinetic properties of ciprofol, which is primarily metabolized through the kidneys rather than the liver, as is the case with propofol (16). This renal metabolism may contribute to the reduced incidence of hypotension and hypoxemia observed in the ciprofol group, as it likely imposes less strain on the cardiovascular and respiratory systems (17). Moreover, the faster recovery time in the ciprofol group suggests that ciprofol could be particularly advantageous in outpatient settings or in procedures requiring quick turnover times, such as FOB (18, 19). The rapid recovery profile of ciprofol could translate into shorter stays in the recovery room, thereby improving patient throughput and reducing the overall cost of care.

The incidence of adverse events in our study also highlights the potential advantages of ciprofol over propofol. Pain on injection, a common and often distressing side effect associated with propofol, was significantly lower in the ciprofol group (20). This reduction in pain is likely due to the lower concentration of the drug in the aqueous phase of the emulsion and the greater hydrophobicity of ciprofol, which may result in a less irritating injection experience (21). This reduction in injection pain likely contributed to the higher patient and physician satisfaction scores observed in the ciprofol group, as pain management is a critical component of overall patient satisfaction during sedation procedures (6). Furthermore, the reduced pain on injection may also have implications for patient willingness to undergo repeat procedures, which is particularly relevant in diagnostic settings like FOB where patients may require multiple follow-up procedures (22). The ability to provide a more comfortable experience during anesthesia induction could enhance patient compliance and reduce anxiety associated with the procedure, potentially leading to better overall outcomes.

## 6 Limitations

Despite the promising results, our study is not without limitations. Firstly, we only included patients with American Society of Anesthesiologists (ASA) classification I-II, excluding those with more severe comorbidities. Consequently, the generalizability of our findings to higher-risk patients (ASA III-IV) remains uncertain (23). This is particularly important because patients undergoing FOB often have significant respiratory or cardiovascular comorbidities, and future studies should aim to include a broader patient population to validate our findings in these higher-risk groups. Additionally, the limited sample size and single-center design of our study may restrict the external validity of the results. Multicenter trials with larger cohorts are necessary to confirm the findings and explore the broader applicability of ciprofol in diverse clinical settings (24).

Secondly, the level of sedation in our study was assessed using subjective scales, which may introduce observer bias. While these scales are commonly used in clinical practice, they are inherently subjective, and future studies should consider incorporating objective measures of sedation depth, such as bispectral index (BIS) monitoring, to provide a more accurate assessment of sedation (25). BIS monitoring could offer a more precise measurement of sedation depth, allowing for better titration of anesthetic agents and potentially reducing the incidence of over- or under-sedation (26). Additionally, while we utilized intermittent injections of ciprofol and propofol in our study, continuous infusion methods could potentially offer more consistent drug levels and may yield different results. Continuous infusion is particularly relevant in longer procedures or in patients who require more prolonged sedation (27). The use of continuous infusion techniques could also reduce the variability in drug plasma concentrations, leading to more stable sedation and fewer fluctuations in hemodynamic parameters (28).

Finally, our study was conducted as a single-center trial with a relatively small sample size, which may limit the generalizability of our results. Larger, multicenter trials are needed to confirm these findings and to explore the potential benefits of ciprofol in a broader clinical context. Additionally, further research should investigate the long-term safety profile of ciprofol, particularly in patients requiring repeated or prolonged sedation, to fully establish its role in clinical practice. The exploration of ciprofol’s interactions with other commonly used medications in anesthesia could also provide valuable insights into its safety and efficacy in more complex clinical scenarios.

In conclusion, this study demonstrates that ciprofol-remifentanil is a promising alternative to propofol-remifentanil for sedation during FOB. Ciprofol not only provides comparable sedation efficacy but also offers several advantages, including improved hemodynamic stability, reduced incidence of injection pain, and higher patient and physician satisfaction. These findings suggest that ciprofol, in combination with remifentanil, could be a valuable anesthesia option for FOB and potentially other similar procedures. However, further research is needed to explore its application in higher-risk patient populations, as well as to optimize dosing regimens, particularly in the context of continuous infusion.

## Data Availability

The original contributions presented in the study are included in the article/supplementary material, further inquiries can be directed to the corresponding author.
